# Fractional-order PI based STATCOM and UPFC controller to diminish subsynchronous resonance

**DOI:** 10.1186/s40064-016-2727-y

**Published:** 2016-09-19

**Authors:** D. Koteswara Raju, Bhimrao S. Umre, Anjali S. Junghare, Mohan P. Thakre, Rambabu Motamarri, Chaitanya Somu

**Affiliations:** 1 Department of Electrical Engineering, VNIT, Nagpur, Maharashtra India; 2 Electrical and Electronics Engineering Department, Aditya Engineering College, Kakinada, Andhrapradesh India

**Keywords:** Fractional-order PI controller, STATCOM, Torque amplification, Voltage source converter, UPFC

## Abstract

This research article proposes a powerful fractional-order PI controller to mitigate the subsynchronous oscillations in turbine-generator shaft due to subsynchronous resonance (SSR) with flexible AC transmission system devices such as static synchronous compensator (STATCOM) and unified power flow controller (UPFC). The diminution of SSR is achieved by the raising of network damping at those frequencies which are proximate to the torsional mode frequency of the turbine-generator shaft. The increase of network damping is obtained with the injection of subsynchronous frequency component of current and both current and voltage into the line. The subsynchronous component of current and voltage are derived from the measured signal of the system and further the same amount of shunt current is injected with STATCOM and simultaneous injection of current and voltage with UPFC into the transmission line to make the subsynchronous current to zero which is the prime source of turbine shaft oscillations. The insertion and proper tuning of Fractional-order PI controller in the control scheme, the subsynchronous oscillations are reduced to 92 % in case of STATCOM and 98 % in case of UPFC as compared to without controller and 14 % as compared with the results of conventional PI controller. The IEEE first benchmark model has adopted for analyze the effectiveness and speed of the proposed control scheme using MATLAB-Simulink and the corresponding results illustrates the precision and robustness of the proposed controller.

## Background

Series capacitor compensation has been broadly employed in Power system to cancel a portion of reactance of the line impedance to increment the power transfer capability of long high voltage (HV) and extra high voltage (EHV) transmission lines further load sharing among parallel lines and boosts the steady state and transient stability limits (Anderson et al. 1989; IEEE SSR Working Group 1985). However, the addition of capacitive compensation in series can cause a new difficulty of turbine-generator shaft oscillations with below the system frequency due to Subsynchronous Resonance (SSR). The subsynchronous oscillations can be excited during the fault or disturbance in the transmission line with series capacitors, when the normal system frequency matches with the complement of any of the mode frequency of the shaft system (Kundur 1994; Padiyar 1998).

Latest advancement of power electronic devices led to the improvement of FACTS devices such as thyristor controlled series capacitor (TCSC), static synchronous compensator (STATCOM), static synchronous series compensator (SSSC) and unified power flow controller (UPFC) (Hingorani and Gyugyi 2000). An astronomically immense number of methods and solutions have been addressed by the different researchers to avoid the problem of SSR with the concern of FACTS devices (Padiyar and Swayam Prakash 2003; Padiyar and Prabhu 2006; Bongiorno et al. 2008b, c; Mohan et al. 2015; Koteswara Raju et al. 2016). The selection of controller is depends on extraction of the subsynchronous frequency components with high speed and accuracy. From the knowledge of subsynchronous frequency component of current and voltage a proper protection system is designed to avoid shaft breakage due to SSR (Bongiorno et al. 2008a).

The mitigation of SSR by the injection of shunt current with STATCOM including proportional integrator controller (PI) is proposed in reference (Umre et al. 2007). The STATCOM with subsynchronous damping controller (SSDC) including type 1 and type 2 controller is proposed based on the tuning of parameters using damping torque method to mitigate the SSR (Padiyar and Swayam Prakash 2003; Padiyar and Prabhu 2006). The parameters of SSDC are tuned to get good performance to provide damping (positive) in the range of torsional mode frequencies. The damping of SSR oscillations with 48 pulses (three-level) VSC based STATCOM using remote signal is proposed in (Salemnia et al. 2008).

The problem of SSR can also be bypassed by a proper combination of hybrid series compensation consists of FACTS controllers (SSSC) along with passive components. The mitigation of SSR with the proper injection of series voltage using PI based SSSC is described in (Bongiorno et al. 2008b, c; Mohan et al. 2015). The injection of subsynchronous frequency component of voltage in series with subsynchronous current suppresser using SSSC based on the knowledge of subsynchronous current to damp out the SSR is proposed in (Panda et al. 2010, 2016;Thirumalaivasan et al. 2013).

In this paper a Fractional-order proportional integrator (FOPI) based STATCOM and UPFC is used for mitigating the subsynchronous oscillations in turbine-generator shaft due to SSR. The mitigation of SSR is done by the injection of shunt current by STATCOM and the simultaneous injection of voltage and current into the line by UPFC with reference to the subsynchronous components which are extracted from the line. The STATCOM and UPFC are constructed with the help of multi-pulse voltage source converters. The fractional-order P I^λ^D^µ^ controller derived from conventional PID controller with integrator of real order λ and differentiator of real order µ. Taking λ = 1 and µ = 1, we obtain a classical PID controller. If µ = 0 and K_d_ = 0, we obtain a P I^λ^ controller, etc. All these types of controllers are particular cases of the P I^λ^D^µ^ controller, which is more flexible and gives the adjustment of dynamical properties of the fractional-order control system. In P I^λ^D^µ^ controller there are five parameters to tune, with respect to the three parameters of the standard PID controller (λ and µ are equal to one) (Igor Podlubny 1999; Shantanu Das 2008). The facility of fine tuning of Fractional-order PI in the control circuit of STATCOM and UPFC controllers the subsynchronous oscillations are reduced to 92 and 98 % as compared to without controller and 14 % as compared to conventional PI controller. Fractional-order PI controllers having larger stability limit with larger phase value as compared to PI controller. Moreover, the Fractional-order PI controllers (FOPI) exhibits a less negative phase than the PI controller and it implies that more robustness (in the sense of stability) to changes in the overall system parameters.

The paper is organized in four sections. “Study system (IEEE FBM) with UPFC controller” section describes UPFC connected IEEE first benchmark model (study system) and the procedure for realization of subsynchronous frequency component of current and voltage. The design of subsynchronous frequency component controller including PI and Fractional-order PI controller and further the study system parameters and specifications of FACTS devices are presented in “Subsynchronous frequency component controller” section. The FFT analysis of LPB-GEN torque signal and simulation results of IEEE first benchmark model without and with FACTS controller under symmetrical (L–L-L) fault using PI and Fractional-order PI controller is given in “Results and discussions” section. The “Conclusion” section represents the conclusion of the complete research work.

## Study system (IEEE FBM) with FACTS controller

**Fig. 1:**
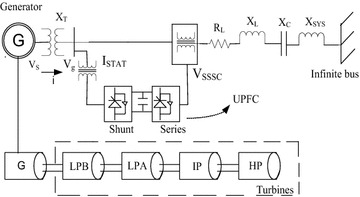
Study system model (IEEE FBM) including UPFC controller

The block diagram of study system (IEEE FBM) with FACTS controller is shown in Fig. 1. The one line diagram of study system with STATCOM is similar as shown in Fig. 1 and only difference is that the STATCOM is connected in shunt to the line. The generated voltage and the grid current are expressed as ${\text{v}}_{\text{s}}$ and ${\text{i}}$ respectively. The injected current and voltage by series and shunt converters are denoted as ${\text{v}}_{\text{SSSC}}$ and ${\text{i}}_{\text{STAT}}$. The main principle of SSR mitigation with classical control scheme is to restore the voltage generated of frequency of fundamental by the added bank of fixed capacitor by injecting a voltage of like magnitude with series converter (Bongiorno et al. 2008b, c; Koteswara Raju et al. 2016). With the injection of shunt current by STATCOM and both current and voltage into the line by UPFC eliminates the capacitive reactance of the capacitor bank and further shifts the system resonance (electrical), thus avoids the problem of subsynchronous resonance. Several publications have described the capability of used control method (Bongiorno et al. 2008b, c; Thirumalaivasan et al. 2013; Mohan et al. 2015; Panda et al. 2010, 2016; Koteswara Raju et al. 2016). The analytical procedure for realization of current and voltage components of subsynchronous frequency from the signal which is measured from the transmission line is proposed as fallows.

### Realization of current and voltage components of subsynchronous frequency

The current and voltage components of below synchronous frequency at the generator terminals are realized by considering the generic case of a transmission line connected with synchronous generator.

The generator voltage in terms of α and β-plane is written as

1 $${\text{v}}_{\text{s}}^{{\left( {\upalpha \upbeta } \right)}} = {\text{v}}_{{{\text{s}},\upalpha}} \left( {\text{t}} \right) + {\text{jv}}_{{{\text{s}},\upbeta}} \left( {\text{t}} \right) =\upomega\left( {\text{t}} \right){\text{V}}_{\text{s}} {\text{e}}^{{{\text{j}}\left( {\upomega_{0} {\text{t}} +\updelta\left( {\text{t}} \right)} \right)}}$$

where V_s_ is the magnitude of terminal voltage of generator at rated speed, phase displacement is given by δ, $\upomega({\text{t}})$ is the speed of rotor in per-unit and $\upomega_{0}$ is the fundamental angular frequency radians/second. The speed of the generator rotor in terms of fundamental angular frequency $\upomega_{0}$, and oscillating angular frequency $\upomega_{\text{m}}$ is given by

2 $$\upomega\left( {\text{t}} \right) =\upomega_{0} + {\text{A}}\sin \left( {\upomega_{\text{m}} {\text{t}}} \right)$$

where A denotes the oscillation magnitude and $\upomega_{\text{m}}$ is the frequency of rotor oscillations.

By applying derivative to the rotor angle

3 $$\frac{\text{d}}{\text{dt}}\updelta\left( {\text{t}} \right) = \left[ {\upomega\left( {\text{t}} \right) -\upomega_{0} } \right]\upomega_{\text{B}} = {\text{A}}\sin \left( {\upomega_{\text{m}} {\text{t}}} \right)\upomega_{\text{B}}$$

where, $\upomega_{\text{B}}$ is the rated frequency in radians/sec. To get $\updelta_{0}$ i.e. the steady state rotor angle, integrate Eq. (3) on both sides. The angle of rotor is expressed as

4 $$\updelta\left( {\text{t}} \right) =\updelta_{0} - {\text{A}}\frac{{\upomega_{\text{B}} }}{{\upomega_{\text{m}} }}\cos \left( {\upomega_{\text{m}} {\text{t}}} \right)$$

When the system subjected to SSR, a small disturbance to generator rotor produces the voltage and current consists of three components: fundamental frequency, bellow synchronous frequency and above synchronous frequency. A little damping of positive is offered by network for super-synchronous frequency; hence the risk offered to generating station by this less (Bongiorno et al. 2008b).

The voltage of subsynchronous frequency component in α and β-plane is given by

5 $${\text{v}}_{{{\text{s}},{\text{sub}}}}^{{\upalpha \upbeta }} \left( {\text{t}} \right) = - \frac{{{\text{AV}}_{\text{s}} }}{{2\upomega_{\text{m}} }}\left( {\upomega_{0} -\upomega_{\text{m}} } \right){\text{e}}^{{{\text{j}}\left[ {\left( {\upomega_{0} -\upomega_{\text{m}} } \right){\text{t}} +\updelta_{0} + \frac{\uppi}{2}} \right]}}$$

Transformed vector of grid voltage in d-q plane is given by

6 $${\text{v}}_{\text{s}}^{\text{dq}} \left( {\text{t}} \right) = {\text{v}}_{\text{s}}^{{\left( {\upalpha \upbeta } \right)}} \left( {\text{t}} \right){\text{e}}^{{ - {\text{j}}\upomega_{0} {\text{t}}}} = {\text{v}}_{{{\text{s}},{\text{f}}}}^{{\left( {\text{dq}} \right)}} \left( {\text{t}} \right) + {\text{v}}_{{{\text{s}},{\text{sub}}}}^{{\left( {\text{dq}} \right)}} \left( {\text{t}} \right)$$

where ${\text{v}}_{{{\text{s}},{\text{f}}}}^{{\left( {\text{dq}} \right)}}$ is the fundamental frequency and the sub-synchronous frequency component in d-q plane is written as

7 $${\text{v}}_{{{\text{s}},{\text{sub}}}}^{{\left( {\text{dq}} \right)}} \left( {\text{t}} \right) = {\text{v}}_{{{\text{s}},{\text{sub}}}}^{{\left( {\upalpha \upbeta } \right)}} \left( {\text{t}} \right){\text{e}}^{{ - {\text{j}}\upomega_{0} {\text{t}}}} = - \frac{{{\text{AV}}_{\text{s}} }}{{2\upomega_{\text{m}} }}\left( {\upomega_{0} -\upomega_{\text{m}} } \right){\text{e}}^{{ - {\text{j}}\left[ {\upomega_{\text{m}} {\text{t}} +\updelta_{0} + \frac{{\uppi }}{2}} \right]}}$$

The terminal voltage in terms of $dq$ co-ordinate system with a small disturbance is given by

8 $${\text{v}}_{\text{s}}^{\text{dq}} \left( {\text{t}} \right) = {\text{v}}_{{{\text{s}},{\text{f}}}}^{\text{dq}} \left( {\text{t}} \right) + {\text{v}}_{{{\text{s}},{\text{sub}}}}^{{\left( {\text{dq}} \right)}} \left( {\text{t}} \right) + {\text{v}}_{{{\text{s}},{ \sup }}}^{{\left( {\text{dq}} \right)}} \left( {\text{t}} \right)$$

**Fig. 2:**
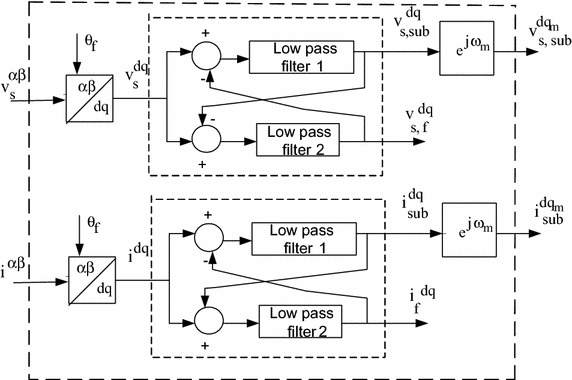
Low pass filter based estimation of subsynchronous components (ESSC)

Figure 2 represents the block diagram of Low Pass Filter (LPF) based estimation of subsynchronous components. ${\text{f}}_{ \sup }$ and ${\text{f}}_{\text{sub}}$ represents the super-synchronous and sub-synchronous frequency component. Consider the generator rotor oscillates with angular frequency $\upomega_{m}$ and the Eq. (7) can be rewritten in $dq_{m}$-plane as

9 $${\text{v}}_{\text{s}}^{\text{dq}} \left( {\text{t}} \right) = {\text{v}}_{{{\text{s}},{\text{f}}}}^{\text{dq}} \left( {\text{t}} \right) + {\text{v}}_{{{\text{s}},{\text{sub}}}}^{{\left( {{\text{dq}}_{\text{m}} } \right)}} \left( {\text{t}} \right){\text{e}}^{{ - {\text{j}}\upomega_{\text{m}} {\text{t}}}}$$

The rearrangement of Eq. (8) gives the extract of sub-synchronous frequency component, so that ${\text{v}}_{{{\text{s}},{\text{f}}}}^{\text{dq}}$ and ${\text{v}}_{{{\text{s}},{\text{sub}}}}^{{\left( {\text{dq}} \right)}}$ become isolated and given to filter (Low pass). The output of Estimation of subsynchronous component (ESSC) is

10 $${\text{v}}_{{{\text{s}},{\text{f}}}}^{\text{dq}} \left( {\text{t}} \right) = {\text{H}}_{\text{f}} \left( {\text{p}} \right)\left[ {{\text{v}}_{\text{s}}^{\text{dq}} \left( {\text{t}} \right) - {\text{v}}_{{{\text{s}},{\text{sub}}}}^{\text{dq}} \left( {\text{t}} \right){\text{e}}^{{{\text{j}}(\upomega_{\text{m}} {\text{t}})}} } \right]$$

11 $${\text{i}}_{{,{\text{f}}}}^{\text{dq}} \left( {\text{t}} \right) = {\text{H}}_{\text{f}} \left( {\text{p}} \right)\left[ {{\text{i }}^{\text{dq}} \left( {\text{t}} \right) - {\text{i}}_{\text{sub}}^{\text{dq}} \left( {\text{t}} \right){\text{e}}^{{{\text{j}}(\upomega_{\text{m}} {\text{t}})}} } \right]$$

12 $${\text{v}}_{{{\text{s}},{\text{sub}}}}^{{{\text{dq}}_{\text{m}} }} \left( {\text{t}} \right) = {\text{H}}_{\text{sub}} \left( {\text{p}} \right)\left[ {{\text{v}}_{\text{s}}^{\text{dq}} \left( {\text{t}} \right){\text{e}}^{{{\text{j}}(\upomega_{\text{m}} {\text{t}})}} - {\text{v}}_{{{\text{s}},{\text{f}}}}^{\text{dq}} \left( {\text{t}} \right)} \right]{\text{e}}^{{{\text{j}}(\upomega_{\text{m}} {\text{t}})}}$$

13 $${\text{i}}_{\text{sub}}^{{{\text{dq}}_{\text{m}} }} \left( {\text{t}} \right) = {\text{H}}_{\text{sub}} \left( {\text{p}} \right)\left[ {{\text{i}}^{\text{dq}} \left( {\text{t}} \right){\text{e}}^{{{\text{j}}(\upomega_{\text{m}} {\text{t}})}} - {\text{i}}_{\text{f}}^{\text{dq}} \left( {\text{t}} \right)} \right]{\text{e}}^{{{\text{j}}(\upomega_{\text{m}} {\text{t}})}}$$

The transfer functions of low pass filter (LPF) are ${\text{H}}_{\text{f}} \left( {\text{p}} \right)$, and ${\text{H}}_{\text{sub}} \left( {\text{p}} \right)$. The subsynchronous frequency component of voltage is written as

14 $${\text{v}}_{{{\text{s}},{\text{sub}}}}^{\text{dq}} \left( {\text{t}} \right) = {\text{H}}_{\text{sub}} \left( {{\text{p}} + {\text{j}}\upomega_{\text{m}} } \right)\left[ {{\text{v}}_{\text{s}}^{\text{dq}} \left( {\text{t}} \right) - {\text{v}}_{{{\text{s}},{\text{f}}}}^{\text{dq}} \left( {\text{t}} \right)} \right]$$

Similarly the subsynchronous frequency component of current is written as

15 $${\text{i}}_{{{\text{s}},{\text{sub}}}}^{\text{dq}} \left( {\text{t}} \right) = {\text{H}}_{\text{sub}} \left( {{\text{p}} + {\text{j}}\upomega_{\text{m}} } \right)\left[ {{\text{i}}_{\text{s}}^{\text{dq}} \left( {\text{t}} \right) - {\text{i}}_{\text{s,f}}^{\text{dq}} \left( {\text{t}} \right)} \right]$$

The voltage component of subsynchronous and fundamental frequency is obtained by combining Eqs. (10) and (14). Equations (11) and (15) are used for the estimation of current component of subsynchronous and fundamental frequency.

## Subsynchronous frequency component controller

To make the current component of subsynchronous to zero, the initiative of the proposed scheme is to generate and inject the internal bus current of subsynchronous with STATCOM and current, voltage by UPFC. The Laplace domain of the sub-synchronous component current controller (SSCC) can be expressed as

16 $${\text{V}}_{{{\text{SSSC}}_{\text{sub}} }}^{{\left( {{\text{dq}}_{\text{m}} } \right)}} \left( {\text{s}} \right) = {\text{v}}_{{{\text{g}},{\text{sub}}}}^{{{\text{dq}}_{\text{m}} }} \left( {\text{s}} \right) + \left( {{\text{R}} + {\text{j}}\left( {\upomega -\upomega_{\text{m}} } \right)\left( {{\text{L}}_{\text{T}} + {\text{L}}^{{\prime \prime }} } \right)} \right) {\text{i}}_{\text{sub}}^{{\left( {{\text{dq}}_{\text{m}} } \right)}} \left( {\text{s}} \right) + \left( {{\text{K}}_{\text{p}} + \frac{{{\text{K}}_{\text{i}} }}{\text{s}}} \right)\left[ {{\text{i}}_{\text{sub}}^{{\left( {{\text{dq}}_{\text{m}} } \right)}} \left( {\text{s}} \right) - {\text{i}}_{\text{sub}}^{{\left( {{\text{dq}}_{\text{m}} } \right)*}} \left( {\text{s}} \right)} \right]$$

17 $${\text{I}}_{{{\text{STAT}}_{{{\text{sub}}}} }}^{{\left( {{\text{dq}}_{{\text{m}}} } \right)}} \left( {\text{s}} \right) = {\text{i}}_{{{\text{s}},{\text{sub}}}}^{{{\text{dq}}_{{\text{m}}} }} \left( {\text{s}} \right) + \left[ {\frac{{{\text{v}}_{{{\text{g}},{\text{sub}}}}^{{\left( {{\text{dq}}_{{\text{m}}} } \right)}} \left( {\text{s}} \right)}}{{\left( {{\text{R}} + {\text{j}}\left( {{\upomega } - {\upomega }_{{\text{m}}} } \right)\left( {{\text{L}}_{{\text{T}}} + {\text{L}}^{{\prime \prime }} } \right)} \right)}}} \right] + \left[ {\left( {{\text{v}}_{{{\text{g}},{\text{sub}}}}^{{\left( {{\text{dq}}_{{\text{m}}} } \right)}} \left( {\text{s}} \right) - {\text{v}}_{{{\text{g}},{\text{sub}}}}^{{\left( {{\text{dq}}_{{\text{m}}} } \right)*}} \left( {\text{s}} \right)} \right)\bigg/\left( {{\text{K}}_{{\text{p}}} + \frac{{{\text{K}}_{{\text{i}}} }}{{\text{s}}}} \right)} \right]$$

where ${\text{R}}$, ${\text{L}}^{{\prime \prime }}$ and ${\text{L}}_{\text{T}}$ are the upstream system resistance with FACTS device, the generator sub transient inductance and transformer leakage inductance respectively. ${\text{i}}_{\text{sub}}^{{\left( {{\text{dq}}_{\text{m}} } \right)*}}$ is the current reference, ${\text{K}}_{i}$ and ${\text{K}}_{\text{p}}$ are the integral and proportional gains of the PI-controller.

### Fractional-order PI (FOPI) controller

The fractional-order P I^λ^ D^µ^ controller was proposed as a generalization of the PID controller with integrator of real order λ and differentiator of real order µ. The transfer function of such type  of controller in Laplace domain has form (Igor Podlubny 1999; Shantanu Das 2008):

18 $${\text{C}}\left( {\text{s}} \right) = \frac{{{\text{U}}\left( {\text{s}} \right)}}{{{\text{E}}\left( {\text{s}} \right)}} = {\text{K}}_{\text{p}} + {\text{K}}_{\text{i}} {\text{s}}^{{ -\uplambda}} + {\text{K}}_{\text{d}} {\text{s}}^{\upmu} ,\quad (\uplambda \cdot\upmu > 0)$$

where K_p_ is the proportional constant, K_i_ is the integration constant and K_d_ is the differentiation constant. Transfer function (18) corresponds in discrete domain with the discrete transfer function in the following expression (Cao and Cao 2006; Enrico Pisoni et al. 2009):

19 $${\text{C}}\left( {{\text{z}}^{ - 1} } \right) = \frac{{{\text{U}}\left( {{\text{z}}^{ - 1} } \right)}}{{{\text{E}}\left( {{\text{z}}^{ - 1} } \right)}} = {\text{K}}_{\text{p}} + {\text{K}}_{\text{i}} \left( {\upomega\left( {{\text{z}}^{ - 1} } \right)} \right)^{{ -\uplambda}} + {\text{K}}_{\text{d}} \left( {\upomega\left( {{\text{z}}^{ - 1} } \right)} \right)^{\upmu} ,\quad (\uplambda,\upmu > 0)$$

where λ and µ are arbitrary real numbers. To get P I^λ^ controller substitute µ = 0 and K_d_ = 0.

With the use of Fractional-order PI controller the Eq. (19) and (20) can be written as

20 $${\text{V}}_{{{\text{SSSC}}_{\text{sub}} }}^{{\left( {{\text{dq}}_{\text{m}} } \right)}} \left( {\text{s}} \right) = {\text{v}}_{{{\text{g}},{\text{sub}}}}^{{{\text{dq}}_{\text{m}} }} \left( {\text{s}} \right) + \left( {{\text{R}} + {\text{j}}\left( {\upomega -\upomega_{\text{m}} } \right)\left( {{\text{L}}_{\text{T}} + {\text{L}}^{{\prime \prime }} } \right)} \right) {\text{i}}_{\text{sub}}^{{\left( {{\text{dq}}_{\text{m}} } \right)}} \left( {\text{s}} \right) + \left( {{\text{K}}_{\text{p}} + \frac{{{\text{K}}_{\text{i}}^{\uplambda} }}{\text{s}}} \right)\left[ {{\text{i}}_{\text{sub}}^{{\left( {{\text{dq}}_{\text{m}} } \right)}} \left( {\text{s}} \right) - {\text{i}}_{\text{sub}}^{{\left( {{\text{dq}}_{\text{m}} } \right) *}} \left( {\text{s}} \right)} \right]$$

21 $${\text{I}}_{{{\text{STAT}}_{{{\text{sub}}}} }}^{{\left( {{\text{dq}}_{{\text{m}}} } \right)}} \left( {\text{s}} \right) = {\text{i}}_{{{\text{s}},{\text{sub}}}}^{{{\text{dq}}_{{\text{m}}} }} \left( {\text{s}} \right) + \left[ {\frac{{{\text{v}}_{{{\text{g}},{\text{sub}}}}^{{\left( {{\text{dq}}_{{\text{m}}} } \right)}} \left( {\text{s}} \right)}}{{\left( {{\text{R}} + {\text{j}}\left( {{{\upomega}} - {{\upomega}}_{{\text{m}}} } \right)\left( {{\text{L}}_{{\text{T}}} + {\text{L}}^{{\prime \prime }} } \right)} \right)}}} \right]\left[ {\left( {{\text{v}}_{{{\text{g}},{\text{sub}}}}^{{\left( {{\text{dq}}_{{\text{m}}} } \right)}} \left( {\text{s}} \right) - {\text{v}}_{{{\text{g}},{\text{sub}}}}^{{\left( {{\text{dq}}_{{\text{m}}} } \right)*}} \left( {\text{s}} \right)} \right)\bigg/\left( {{\text{K}}_{{\text{p}}} + \frac{{{\text{K}}_{{\text{i}}}^{{{{\uplambda}}}} }}{{\text{s}}}} \right)} \right]$$

**Fig. 3:**
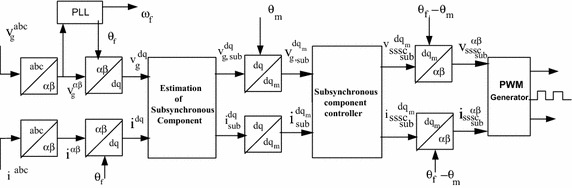
Schematic diagram of the subsynchronous frequency component controller

The Fractional-order PI controller provides the facility for fine tuning of controller to achieve better result as compare to conventional PI controller. With the facility of choosing the appropriate K_p_, K_i_ and λ values, the fine tuning of controller reduces the SSR oscillations with a faster rate. The complete block diagram of subsynchronous frequency component controller is shown in Fig. 3. At First the current and voltage of three-phase are measured from transmission line and further converted into $\alpha \beta$ –plane, with the help of $\theta_{f}$(transformation angle) again converts into to $dq$-coordinate system. The outcome of estimation unit is the subsynchronous and fundamental frequency component of current and voltage in $dq$-frame. The subsynchronous frequency component in $dq$-reference is converted into subsynchronous frequency ${\text{dq}}_{\text{m}}$-frame with the help of $\uptheta_{\text{m}}$ (transform angle) which is obtained by integrating $\upomega_{m}$(oscillating frequency). The consequential signals are given to the subsynchronous frequency component controller (SSCC).

The output of Subsynchronous component controller is again transferred into $\upalpha \upbeta$ and further transferred to abc and are furthermore given to the Pulse width modulation (PWM) generator which issues firing signals to 3-phase 48 pulse (three-level) GTO based voltage source converters (VSC).

### Parameters and specifications of IEEE first benchmark model

To investigate the capability of the proposed control scheme the well-known IEEE FBM with five mass systems has considered and the parameters of the system are shown in Tables 1, 2 and 3. The turbine-generator set of rating 892.4 MVA connected through radial compensated (series) line to an infinite bus and the corresponding voltage is 539 kV of 60 Hz frequency. A Matlab code is developed to outline the natural frequencies of turbine and mode shapes (Anderson et al. 1989; Kundur 1994). The natural frequencies are 1.8002, 16.1335, 24.4785, 32.237, and 47.4563 Hz. For 55 % series compensation the resonant frequency is 28.48 Hz (Kundur 1994).

**Table 1:** IEEE First benchmark network parameters

Resistance of network	${\text{R}}_{\text{L}}$	0.0113 per unit
Reactance of transformer	${\text{X}}_{\text{T}}$	0.142 per unit
Transformation ratio		22 kV/539 kV
Reactance of line	${\text{X}}_{\text{L}}$	0.50 per unit
Reactance of transmission line	${\text{X}}_{\text{sys}}$	0.080 per unit

**Table 2:** Synchronous machine parameters

Parameter	Value (per unit)	Time constant	Value (sec)
${\text{X}}_{\text{a}}$	0.130	${\text{T}}_{{{\text{d}}0}}^{ '}$	4.3
${\text{X}}_{\text{d}}$	1.79	${\text{T}}_{{{\text{d}}0}}^{''}$	0.032
${\text{X}}_{\text{d}}^{ '}$	0.169	${\text{T}}_{{{\text{q}}0}}^{ '}$	0.85
${\text{X}}_{\text{d}}^{''}$	0.135	${\text{T}}_{{{\text{q}}0}}^{''}$	0.05
${\text{X}}_{\text{q}}$	1.71		
${\text{X}}_{\text{q}}^{ '}$	0.228		
${\text{X}}_{\text{q}}^{''}$	0.200

**Table 3:** IEEE first benchmark shaft parameters

Inertia	${\text{H }}\left[ {{\text{s}}^{ - 1} } \right]$	Shaft section	Spring constant [per unit T/rad]
High pressure turbine	0.092897	HP-IP	19.303
Intermediate pressure turbine	0.155589	IP-LPA	34.929
Low pressure A turbine	0.858670	LPA-LPB	52.038
Low pressure B turbine	0.884215	LPB-GEN	70.858
Generator	0.868495		

The selection of tuned values for the parameters of PI controller is given in (Bongiorno et al. 2008c) and the FOPI controllers are given in Table 4.

**Table 4:** Parameters of PI and FOPI controllers

Controllers	${\text{K}}_{\text{p}}$	${\text{K}}_{\text{i}}$	λ
PI	2.6	8	1
FOPI	1.2	2	0.82

### Rating of shunt and series converters of UPFC

A three-phase 48 pulse (three-level) VSC bridge is used for Shunt and Series Converters. The VSC characterized in this research work is a harmonic neutralized 48-pulse GTO based inverter. These arrangements are made to produce harmonic free voltage output by proper connecting 6-pulse VSC’s. 12-pulse configuration is achieved by connecting two 6-pulse VSC’s, two 12-pluse converter are used for a 24-pulse topology and two 24-pulse arrangements are used for obtain a 48-pulse VSC. To produce a 48-pulse waveform with a harmonic content of n = 48 m ± 1, where m = 0, 1, 2, …, the 6-pulse converters requires relative phase displacements accomplished via the gate pulse pattern that determines the angle of the resulting three phase output voltages. Also, PST’s are used and are connected in serially with the phase voltages in the primary side of the MCC transformers to add voltage components in quadrature. These quadrature voltages are obtained from the three phase output voltages of each VSC (Kumar and Ghosh 1999; Hingorani and Gyugyi 2000; Salemnia et al. 2008). The rating of power necessary to diminish the SSR is difficult to conclude and depends on level of compensation (series), duration and location of fault. As the amount of current and voltage components of sub-synchronous frequency is reduced, the Shunt and Series converter rating is also reduced (0.1–5 %). The power rating is 12 MVA and the voltage rating is 8 kV (either capacitor or Direct current source). For an active power of 0.5 pu, the results are obtained and analyzed in the paper.

## Results and discussions

### FFT analysis

**Fig. 4:**
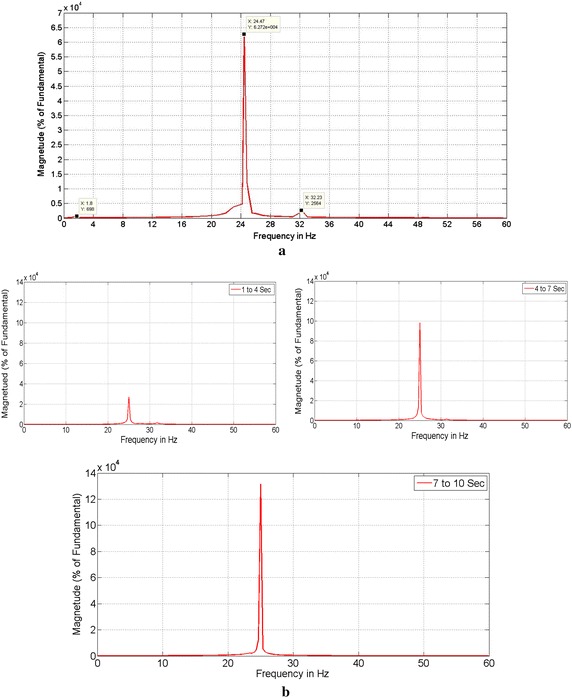
The FFT analysis of rotor speed and LPB-Gen Torque signal without controller. **a** Determination of dominant mode with FFT analysis on rotor speed signal of generator and **b** FFT analysis on LPB-Gen Torque signal without controller

To assess the oscillatory modes of IEEE First Benchmark model, Fast Fourier Transform (FFT) analysis has been made on rotor speed and the corresponding response is shown in Fig. 4a. With 55 % series compensation the electrical resonance frequency coincides with mode 2 of the IEEE FBM, for this the system is unstable. By observing Fig. 4a, there are three modes are present in which mode 2 (24.478 Hz) is more dominant than other two. The torque amplification effect between LPB and Generator for a time interval from 0 to 10 s with division of 3 s in order to clearly observe the dominant mode of SSR and the corresponding results of FFT based analysis without controller is shown in Fig. 4b. The dominant mode (Mode-2) component increases with time as shown in Fig. 4b. Hence there is a requirement of controller to mitigate this adverse, oscillatory and increasing component of instability in the rotor shaft so as to protect the power system from damage.

**Fig. 5:**
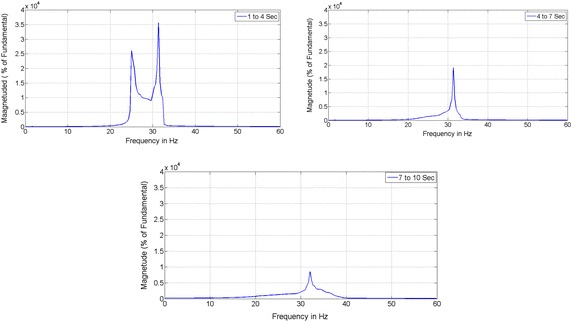
FFT analysis on LPB-Gen Torque signal with controller

Figure 5 shows the FFT analysis of LPB and Generator torque signal with controller.

The subsynchronous mode with frequency of 24.8 Hz is reduced (mitigated) with the addition of controller and a small value is at 32.23 Hz which is not harm to the system is shown in Fig. 5.

### Stress analysis

Transient disturbance in the generator produces accelerating torque (T_a_). Due to this torque, varying stress is produces in the rotor shaft. When this stresses exceeds the endurance limit i.e. 45 × 10^7^ N/m^2^, the shaft will be damaged. The torsional fatigue of turbine generator shaft is primarily as a function of amplitude of the stress and secondarily on its limit (Kundur 1994). The calculation of mechanical stress needs the calculation of mechanical angle (twist angle between shafts) δ and is as fallows.

22 $$\left[ {\text{M}} \right]\left[ {{\ddot{\delta }}} \right] + \left[ {{\text{D}}^{{\prime }} } \right]\left[ {{\dot{\delta }}} \right] + \left[ {\text{K}} \right]\left[\updelta \right] = \left[ {{\text{T}}_{\text{m}} } \right] - \left[ {{\text{T}}_{\text{e}} } \right]$$

where, [M] is diagonal matrix, consisting of inertia of all masses, $\left[ {{\text{D}}^{{\prime }} } \right]$ is tri diagonal symmetric matrix consisting of, various damping coefficients of the masses, [K] is tri diagonal symmetric matrix consisting of torsional stiffness of various mass sections, [T_m_] consists of mechanical torque acting on various masses, [T_e_] consists of electrical torque produced by various masses.

The mechanical stress or Fatigue between any shaft systems is given as

23 $${\text{F}} = \left\{ {\left( {\updelta_{\text{i}} -\updelta_{{{\text{i}} + 1}} } \right) \times {\text{G}} \times {\text{R}}} \right\}/{\text{L}}\quad \left( {{\text{where}}\;{\text{i}} = 1\;{\text{to}}\;5} \right)$$

where F: Fatigue or Mechanical stress in N/m^2^, δ_i_, δ_i+1_: twist angle of ith mass to the (i + 1)th mass in radians, G: modulus of rigidity in N/m^2^; L: length of shaft in meters; R: radius of shaft in meters.

### Simulation results

To realize the performance of the suggested control scheme to diminish the SSR due to Torque Amplification, the IEEE FBM with STATCOM and UPFC has been simulated using Matlab-Simulink software. A three-phase fault is applied to the grid at 1 s for time duration of 0.05 s with 55 % series compensation. The simulation results are shown for three cases, without controller, with PI and with FOPI based STATCOM and UPFC controller. After the clearance of fault at 1.05 s the system has to regain its previous state that is the turbine-generator shaft oscillations are at normal level.

**Fig. 6:**
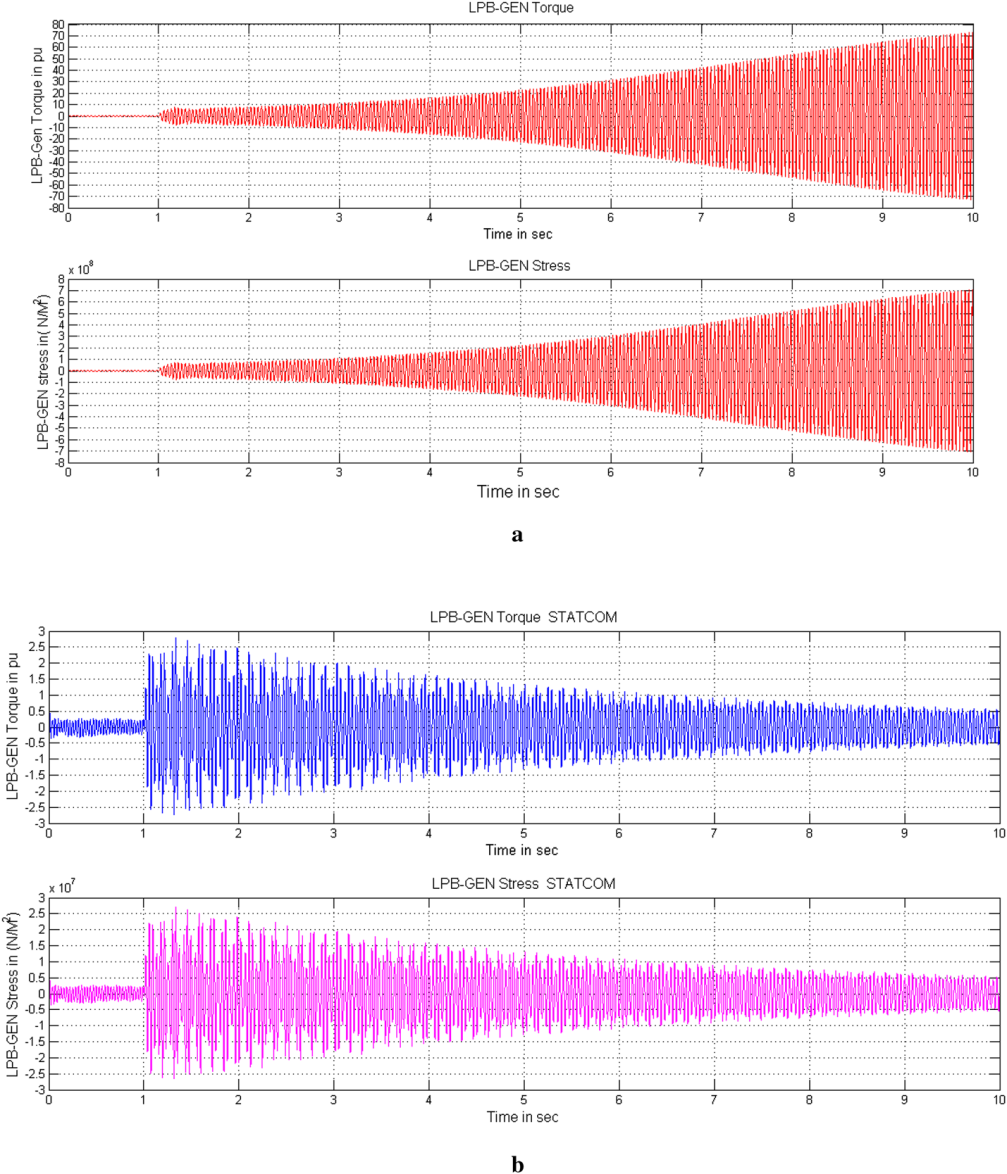
Simulated Torque and Stress between LPB-Generator of IEEE first benchmark model without controller and with PI based STATCOM controller. **a** Without controller and **b** With PI based STATCOM controller

**Fig. 7:**
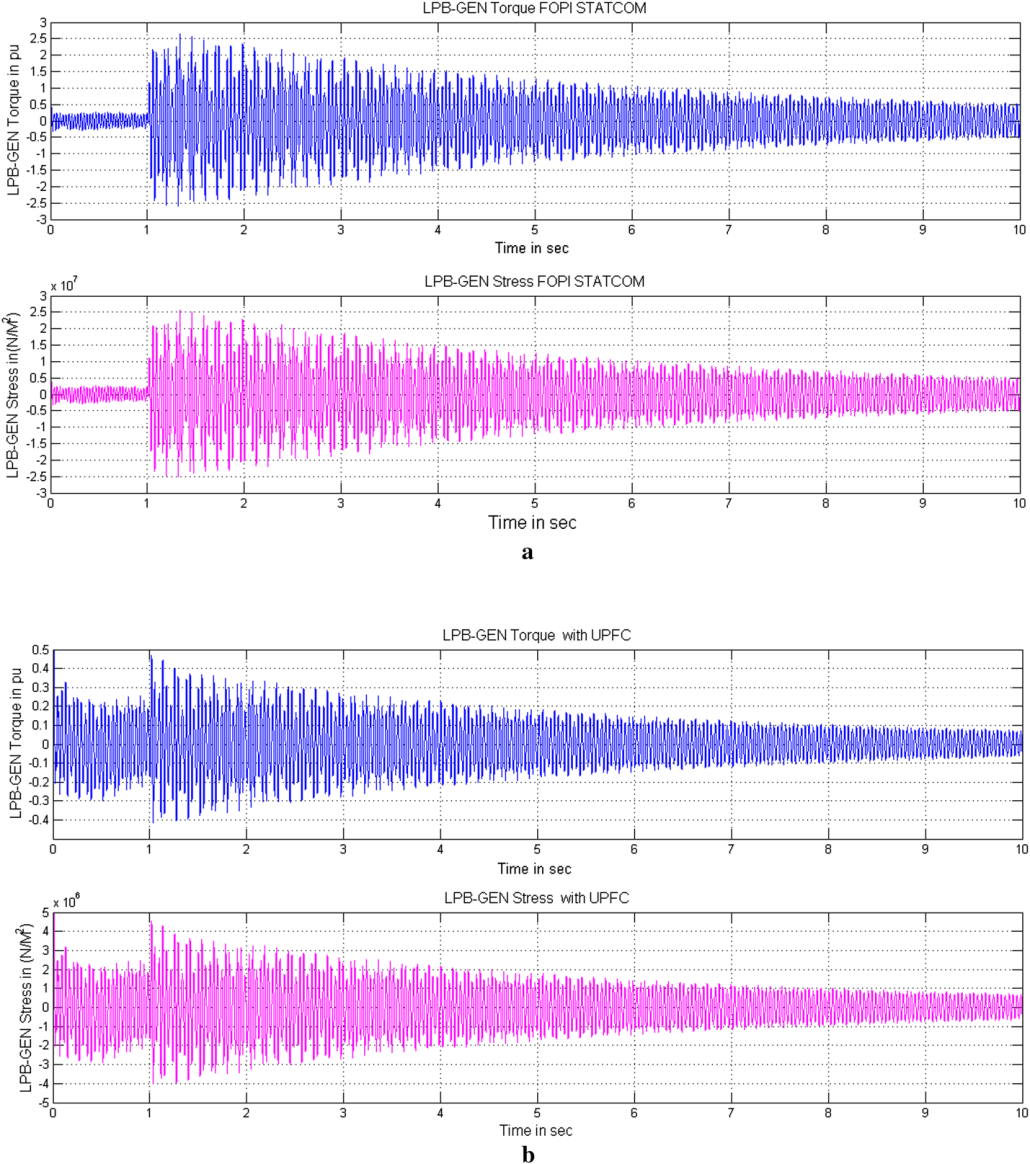
Simulated Torque and Stress between LPB-Generator of IEEE first benchmark model with FOPI based STATCOM and PI based UPFC controllers. **a** With FOPI based STATCOM controller and **b** With PI based UPFC controller

**Fig. 8:**
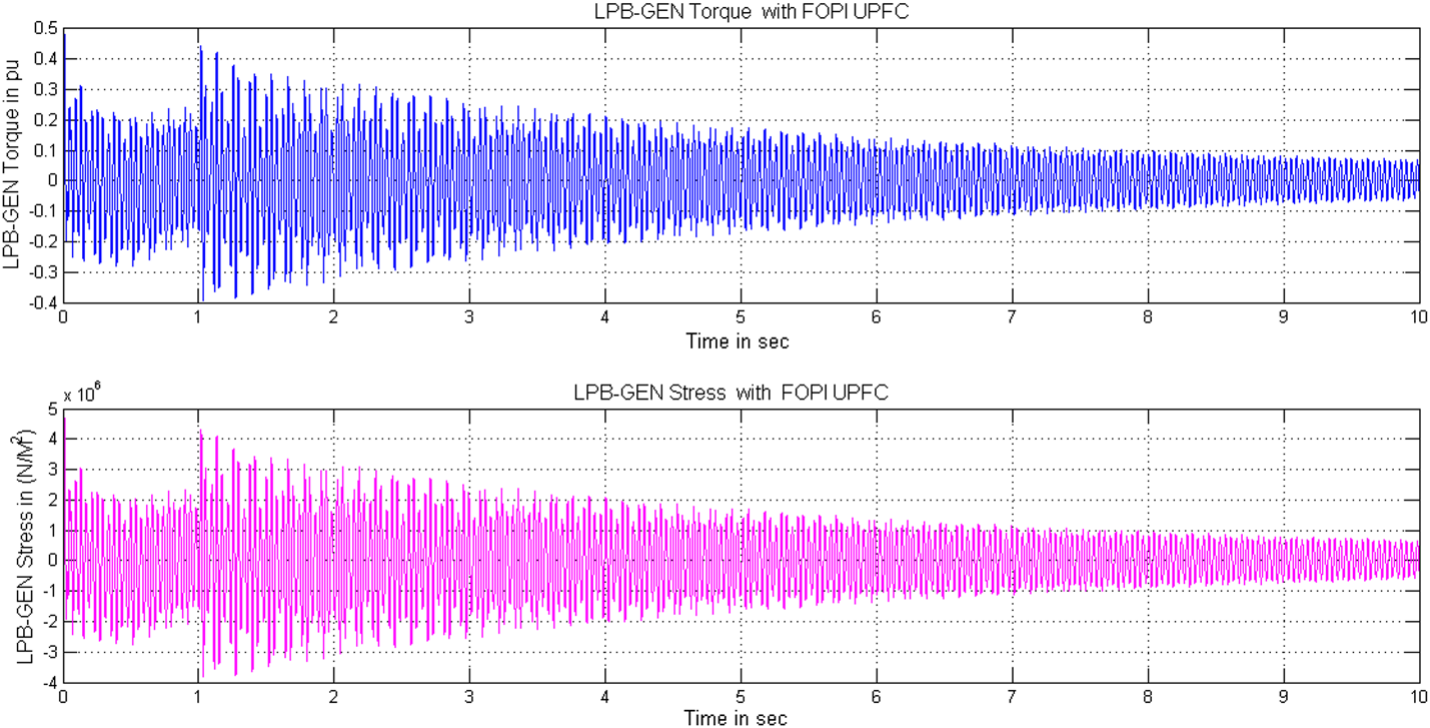
Simulated Torque and Stress between LPB-Generator of IEEE first benchmark model with FOPI based UPFC controller. With FOPI based UPFC controller

Under subsynchronous resonance condition the system is very sensitive, further a small fault or disturbance causes large amplification of turbine-generator oscillations and furthermore increases the stress and twist between the shaft sections that will damage the entire system. Figures 6, 7 and 8 show the simulated Torque and Stress between LPB-Generator shaft without controller and with STATCOM and UPFC controller. Due to the unstable mode, when the fault is cleared, large oscillations will be experienced between the different sections of the turbine-generator shaft. Figure 6a shows the torque and stress between LPB and Generator without controller. For the sake of simplicity the torque and stress between remaining shaft systems experience the same and are not shown. The torque and stress increases with a fast rate after fault clearance which will damage the turbine shaft system. The torque between LPB and Generator increases to 75 times and the stress increases to 75 × 10^7^ N/m^2^ which will damage the entire shaft system. To avoid the damage due to torque amplification effect of SSR the PI controller based STATCOM or UPFC is connected to the line. The PI based STATCOM injects a shunt current or simultaneous injection of shunt current and series voltage with UPFC into the line in order to reduce the capacitive reactance from the capacitor bank and further shift the electrical resonance of the system, thus avoiding the risk of SSR. The simultaneous injection of shunt current and series voltage with UPFC, the turbine oscillations and stress are reduced to such a low value about 94 % as compared to without controller. With PI based STATCOM controller the torque between LPB and Generator is reduced from 2.2 to 0.3 pu and further reduced from 0.46 to 0.09 pu in case of UPFC. Similarly the Mechanical stress between LPB and Generator is reduced from 2.2 × 10^7^ to 0.4 × 10^7^ N/m^2^ with STATCOM and 0.45 to 0.06 × 10^7^ N/m^2^ with UPFC for a time interval of 10 s shown in Figs. 6b and 7b. With the facility of fractional tuning of FOPI controller the torque between LPB and Generator is further reduced from 2.1 to 0.28 pu in case of STATCOM and from 0.42 to 0.08 pu with UPFC. Similarly the Mechanical stress is furthermore reduced from 2.1 × 10^7^ to 0.34 × 10^7^ N/m^2^ in case of STATCOM and from 0.42 × 10^7^ to 0.05 × 10^7^ N/m^2^ in case of UPFC for a time interval of 10 s shown in Figs. 7a and 8.

**Fig. 9:**
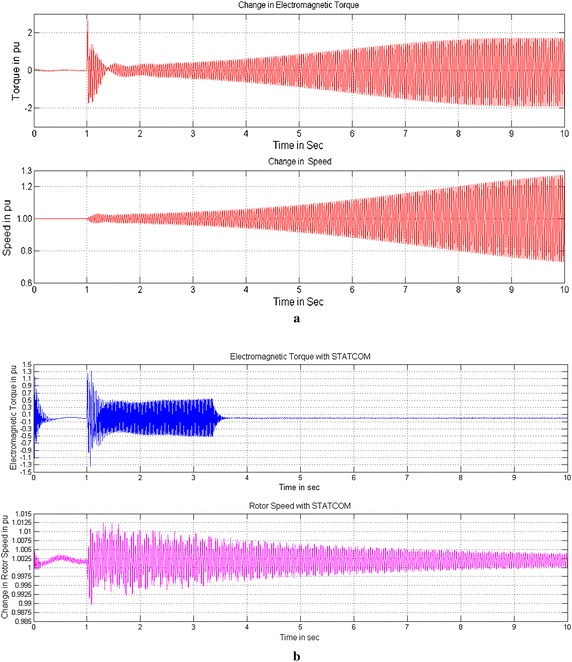
Change in Electro-Magnetic torque and change in Rotor speed of IEEE first benchmark model without controller and with PI based STATCOM controller. **a** Without controller and **b** With PI based STATCOM controller

**Fig. 10:**
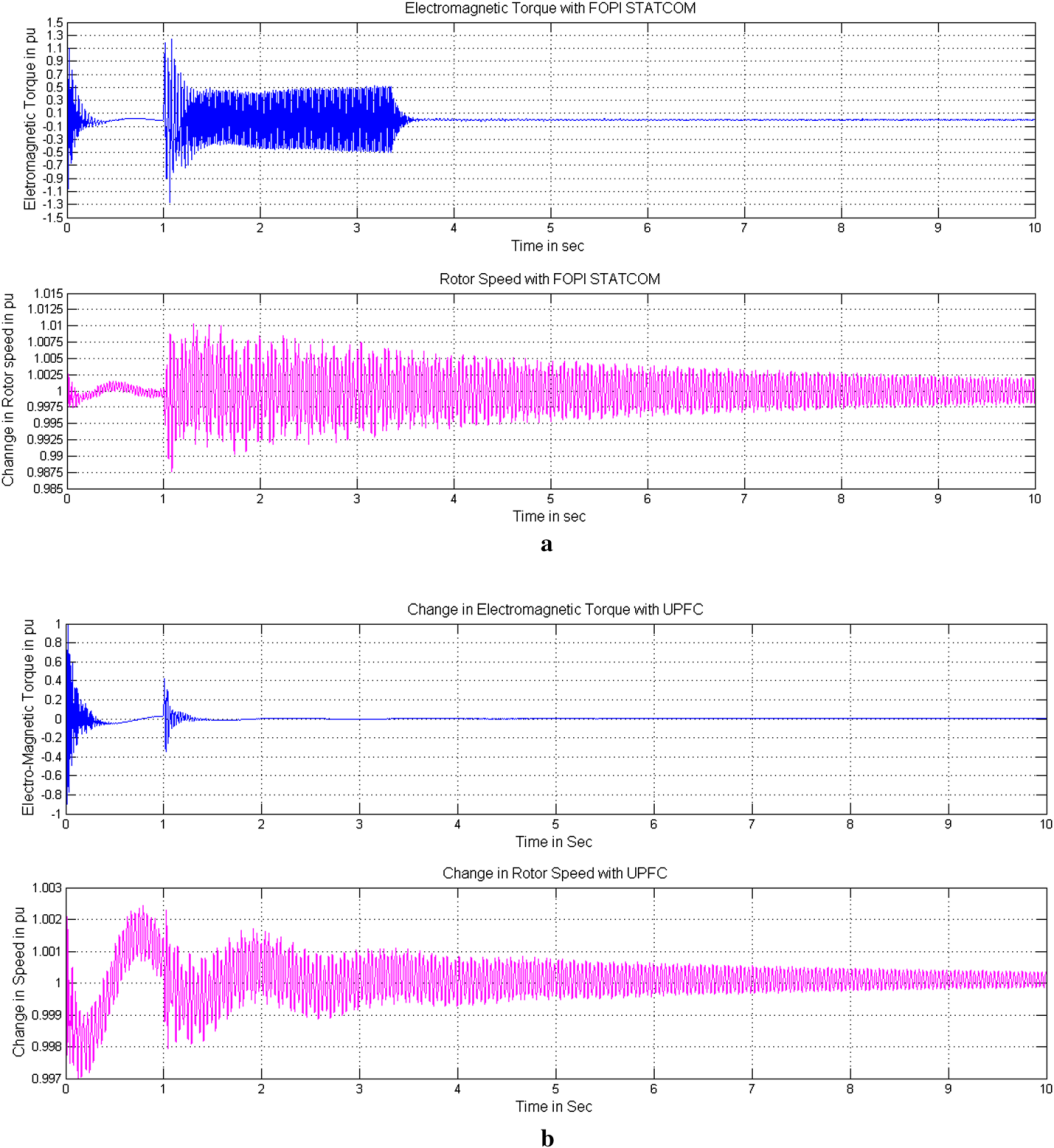
Change in Electro-Magnetic torque and change in Rotor speed of IEEE first benchmark model with FOPI based STATCOM and PI based UPFC controllers. **a** With FOPI based STATCOM controller and **b** With PI based UPFC controller

**Fig. 11:**
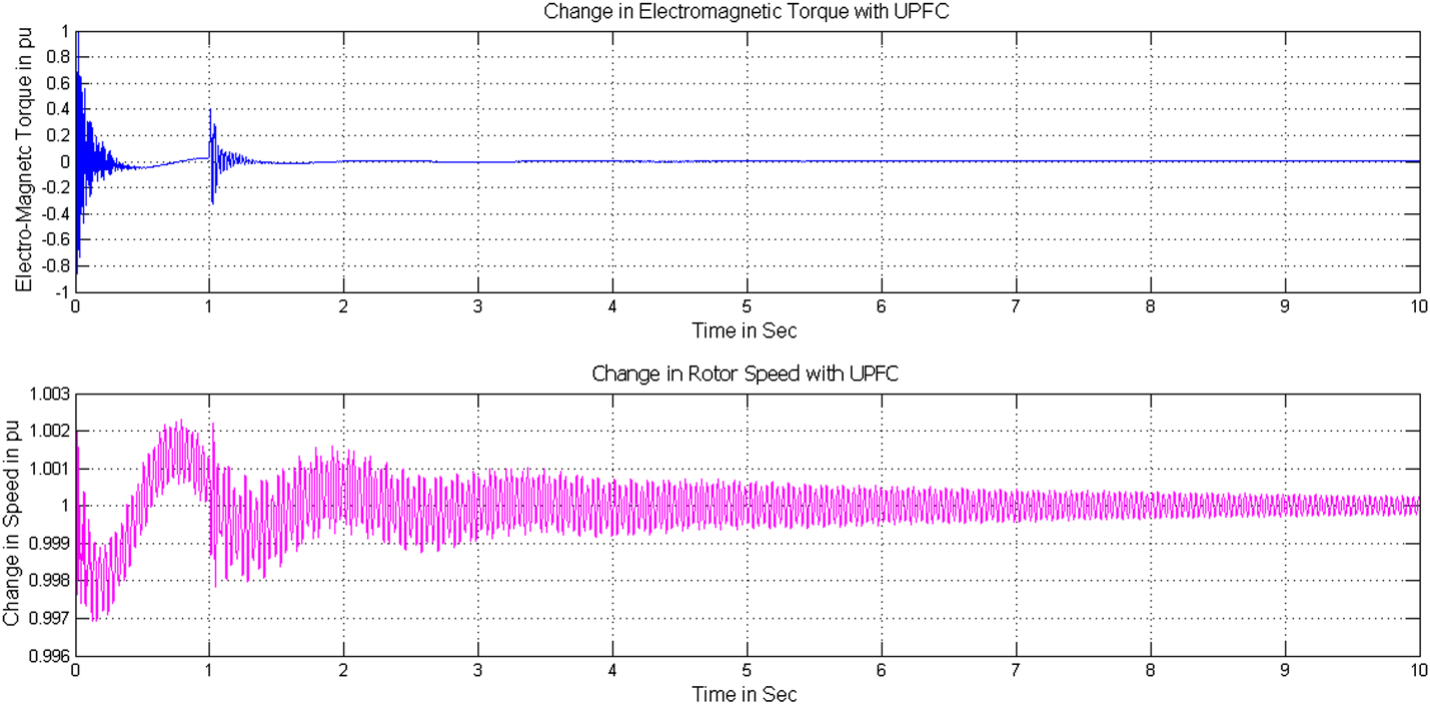
Change in Electro-Magnetic torque and change in Rotor speed of IEEE first benchmark model with FOPI based UPFC controller. With FOPI based UPFC controller

Figures 9, 10 and 11 show the change in Electromagnetic torque and Rotor speed without and with controllers. Without controller both are increasing drastically will lead to shaft damage of Turbine-Generator set shown in Fig. 9a. The Electromagnetic torque increases to 1.8 times and the rotor speed also increases to 1.28 times which is beyond the tolerable limit of Turbine-Generator Shaft system. Figures 9b and 10b show the reduction of change in Electromagnetic torque to a safe value from 0.9 to 0.05 and 0.27 to 0.01 pu and similarly the change in Rotor speed from 1.008 to 1.002 and 1.0015 to 1.001 pu with PI based STATCOM and UPFC controllers for a time interval of 10 s. With the use of FOPI instead of PI controller the change in Electromagnetic torque is further reduced from 0.86 to 0.04 and 0.25 to 0.01pu and the change in Rotor speed are also reduced from1.0077 to1.0018 pu and 1.0012 to 1.001 in case of STATCOM and UPFC for a time interval of 10 s shown in Figs. 10a and 11.

By considering the Figs. 9b, 10a, b and 11 the conclusion is that both the controllers reduce the effect of SSR on Electromagnetic torque and Rotor speed and further the FOPI based controllers are more effective than conventional PI based controllers.

**Fig. 12:**
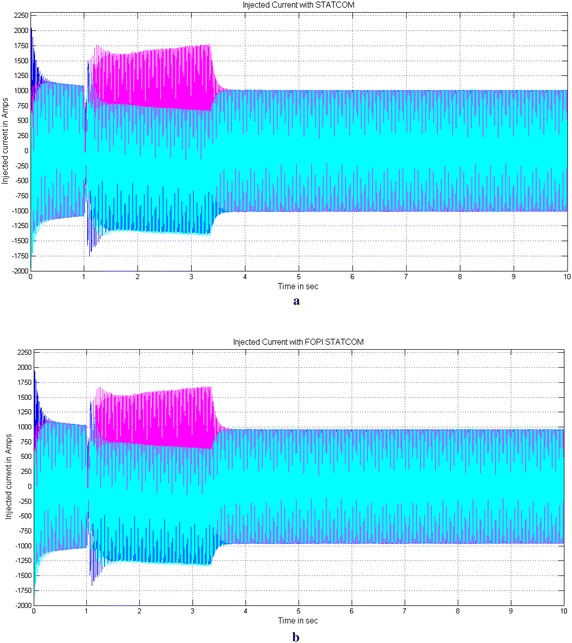
Injected currents of PI and FOPI based STATCOM controllers during three-phase fault to mitigate the SSR. **a** Current injected by PI based STATCOM controller and **b** Current injected by FOPI based STATCOM controller

**Fig. 13:**
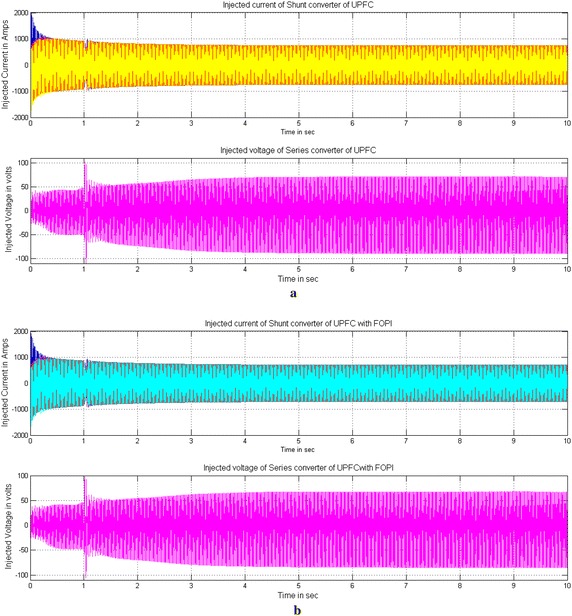
Current and voltage injected by PI and FOPI based UPFC controllers during three-phase fault to mitigate the SSR. **a** Shunt current and series voltage injected by PI based UPFC controller and **b** Shunt current and series voltage injected by FOPI based UPFC controller

Figures 12 and 13 show the Current injected by STATCOM and simultaneous injection of current and voltage by UPFC controllers during three-phase fault to mitigate the SSR with PI and FOPI controllers. The magnitude of injection current by PI based STACOM is 1700 A and the magnitude of simultaneous injection of current and voltage with PI based UPFC is approximately 600 A and 100 V during fault period shown in Figs. 12a, 13a. The magnitude of injected current of STATCOM is reduced to 1600 A and simultaneous injection of current and voltages of UPFC is reduced to approximately 550 A and 60 V during fault period with FOPI based controller shown in Figs. 12b, 13b respectively.

Due to reduction of injected current by STATCOM and simultaneous injected current and voltage by UPFC the burden on converters are further reduced. This is the additional achievement of the proposed Fractional-order PI control scheme. Table 5 gives the comparison of various performance indexes of IEEE first bench mark model with PI and FOPI based STATCOM and UPFC. The table gives the complete idea of reduction of various performance indexes of IEEE first benchmark model with PI and FOPI based controllers for a simulation time interval of 10 s.

**Table 5:** Comparison of different performance indexes of IEEE first benchmark model with PI and with FOPI based STATCOM and UPFC controllers

1. LPB-GEN Torque (per unit) With PI based controller the torque is reduced from 2.2 to 0.3 pu in case of STATCOM and from 0.46 to 0.09 pu with UPFC for a time interval of 1.05 s to 10 s. The torque is further more reduced with FOPI controller from 2.1 to 0.28 pu in case of STATCOM and from 0.42 to 0.08 in case of UPFC	
2. LPB-GEN Stress (10^7^ N/m^2^) The Mechanical stress between LPB and Generator is reduced from 2.2 × 10^7^ to 0.4 × 10^7^ N/m^2^ and 0.45 to 0.06 × 10^7^ N/m^2^ with PI based STATCOM and UPFC for a time interval of 1.05 to 10 s. Much more reduction of stress is obtained with the help of FOPI based controller from 2.1 × 10^7^ to 0.34 × 10^7^ N/m^2^ and from 0.45 to 0.05 × 10^7^ N/m^2^ in case of STATCOM and UPFC	
3. Change in Electro-Magnetic Torque (per unit) The Electromagnetic torque is reduced and reaches to a safe value from 0.9 to 0.05 and 0.27 to 0.01 pu with PI based STATCOM and UPFC controllers for a time interval of 1.05 to 10 s. With the use of FOPI in place of PI controller the change in Electromagnetic torque is further reduced from 0.86 to 0.04 pu and 0.25 to 0.01pu in case of STATCOM and UPFC	
4. Change in Rotor Speed (per unit) Without SSR controller the rotor speed is changes from 1.05 to 1.28 times. The change in Rotor speed is reduced from 1.008 to 1.002 and 1.0015 to 1.001 pu with PI based STATCOM and UPFC controllers for a time interval of 1.05 to 10 s. The use of FOPI in place of PI controller the change in Rotor speed is further reduced from1.0077 to1.0018 pu and 1.0012 to 1.001 with STATCOM and UPFC	

By observing the Table 5 the FOPI based UPFC controller is more effective and faster in mitigation of Torque amplification due to SSR as compared to PI based STATCOM, UPFC and FOPI based STATCOM controllers.

## Conclusion

In this work, a robust Fractional-order PI based STATCOM and UPFC controllers are developed to diminish the oscillations in turbine-generator shaft due to torque amplification effect of SSR. Based on the control system the mitigation of subsynchronous resonance is achieved by raising the damping of network with proper estimation and injection of subsynchronous component quantities into the line using UPFC and the results are compared with STATCOM controller. For the system studied, the superiority of proposed FOPI based controller is demonstrated by comparing the results of conventional PI controller for all cases. From the proposed study, it was observed that, fine and fast SSR mitigation is achieved by Fractional-order PI controller based UPFC as compared to conventional PI based STATCOM, UPFC and Fractional order PI based STATCOM controllers. Moreover, the performance of proposed controller is evaluated under three-phase fault with different performance indices namely; torque and mechanical stress between LPB-Generator, change in electromagnetic torque and change in rotor speed.
